# Association of Late Life Depression, (Non-) Modifiable Risk and Protective Factors with Dementia and Alzheimer’s Disease: Literature Review on Current Evidences, Preventive Interventions and Possible Future Trends in Prevention and Treatment of Dementia

**DOI:** 10.3390/ijerph17207475

**Published:** 2020-10-14

**Authors:** Chih-Yun Kuo, Ivo Stachiv, Tomas Nikolai

**Affiliations:** 1 Department of Neurology and Centre of Clinical Neuroscience, First Faculty of Medicine, Charles University, 12108 Prague, Czech Republic; tomas.nikolai@lf1.cuni.cz; 2 Institute of Physics, Czech Academy of Sciences, 18221 Prague, Czech Republic; 3 Drážní revize s.r.o., Místecká 1120/103, 70300 Ostrava-Vitkovice, Czech Republic

**Keywords:** dementia, Alzheimer’s disease, late life depression, apolipoprotein E, testosterone, obesity, social engagement, substance abuse

## Abstract

The number of people living with dementia and Alzheimer’s disease is growing rapidly, making dementia one of the biggest challenges for this century. Many studies have indicated that depression plays an important role in development of dementia, including Alzheimer’s disease; depression, especially, during the late life may either increase the risk of dementia or even being its prodromal stage. Despite a notably large number of carried observational studies and/or clinical trials, the association between the late life depression and dementia remains, due to the complexity of their relationship, still unclear. Moreover, during past two decades multiple other (non-)modifiable risk and possibly protective factors such as the hypertension, social engagement, obesity, level of education or physical (in)activity have been identified and their relationship with the risk for development of dementia and Alzheimer’s disease has been extensively studied. It has been proposed that to understand mechanisms of dementia and Alzheimer’s disease pathogeneses require their multifactorial nature represented by these multiple factors to be considered. In this review, we first summarize the recent literature findings on roles of the late life depression and the other known (non-)modifiable risk and possibly protective factors in development of dementia and Alzheimer’s disease. Then, we provide evidences supporting hypotheses that (i) depressive syndromes in late life may indicate the prodromal stage of dementia (Alzheimer’s disease) and, (ii) the interplay among the multiple (non-)modifiable risk and protective factors should be considered to gain a better understanding of dementia and Alzheimer’s disease pathogeneses. We also discuss the evidences of recently established interventions considered to prevent or delay the prodromes of dementia and provide the prospective future directions in prevention and treatment of dementia and Alzheimer’s disease using both the single-domain and multidomain interventions.

## 1. Introduction

Life expectancy that doubled in the past two centuries, and is often considered one of the major achievements of mankind in modern history. This achievement, contributed by fast progress in medicine, has not only helped increase longevity, but also brought new challenges to society and national health care systems due to the increased number of people living with cardiovascular diseases [1,2], cancer [2] or depression [3,4,5]. Depression, particularly in late life, is highly prevalent and is commonly believed being a risk factor for development of dementia [6,7,8,9,10]. Psychiatrists expect the number of people with dementia to almost triple over the next 30 years [11,12].

Deaths related to Alzheimer’s disease (AD), the most common (~60% cases) and the costliest to the national health care systems type of dementia, have increased by more than 100% in the past decade [11]. Majority of people with AD suffer with other complications such as cardiovascular diseases [12,13] or diabetes mellitus [14,15]; therefore, they may require longer stays in hospitals equipped with advanced nursing facilities and specially trained stuff. The expected further growth of people with AD would have a strong impact on society, making AD one of the biggest global challenges of the 21st century [16]. Despite the enormous efforts for finding a cure, AD is still a non-preventable and irreversible form of dementia causing cognitive and functional changes that, in turn, impair individuals’ daily life functioning.

During the past few decades researchers have identified multiple other (non-)modifiable risk and protective factors. Since the early stage diagnoses with the relevant interventions may help to significantly delay the prodromes of dementia [17,18,19,20], therefore the association of the other (non-) modifiable risk and protective factors with dementia has been also extensively studied. In addition, there is an increasing amount of evidence supporting the relationship between a “healthy” lifestyle (e.g., physical activities and social engagement) and a reduced risk for the development of dementia in later life [21,22,23]. We emphasize here that the healthy lifestyle is of great importance because dementia and AD are both the heterogeneous disorders caused by a combination of the multiple (non-)modifiable factors and, as such, the oldest-old people (80+) often have mixed dementia, very complicated to treat [21].

The aim of this review is to provide a literature overview of the most recent findings on roles of each of the already identified (non-)modifiable risk and possibly protective factors in the risk for development of dementia and AD. We first discuss the current understanding of how the late life depression may be associated with dementia. Then, in the following section the relationships among the other (non-)modifiable risk factors, possibly protective factors, and dementia (AD) including the currently considered interventions and the foreseen studies are given. Finally, in the last part of this review, we briefly summarize the prospective future trends and directions in the prevention, treatment and research of dementia and AD using single-domain and multidomain interventions.

## 2. Methods

We remind the reader that in contrast to a systematic review, where an exhaustive (complete) summary of the recent literature to a very specific question or questions is given, the purpose of present review is to provide the available and most relevant evidence associating the (non-) modifiable risk factors with development of dementia and AD. This review, therefore, does not answer more specific questions related to the relationship among the (non-)modifiable risk factors and development of dementia and AD (e.g., whether the positive air pressure treatment of sleep disorders can affect the progress of AD [24]). The literature search was performed among the main worldwide electronic databases, that is, PubMed, Google Scholar, Scopus and the Cochrane Library. Importantly, the search criteria and screening of potential studies were in accordance with the PRISMA extension for scoping review [25]. As a result, the following key terms mainly in the form of the corresponding medical subject heading term (MeSH) were searched: “Alzheimer” or “Alzheimer’s disease” or “AD” or “dementia” or “vascular dementia” or “Lewy body dementia” or “dementia/prevention and control” and “depression” or “non-modifiable risk factor” or “risk factor” or “modifiable risk factor” or “protective factor” or “apolipoprotein E (APOE)” or “APOE gene” or “sex hormones” or “estrogen” or “testosterone” or “obesity” or “hypertension” or “social engagement” or “diabetes mellitus” or “education” or “alcohol” or “smoking” or “drugs” or “sleep disturbance” or “obstructive sleep apnea”, “eHealth”. 

The inclusion criteria for this study were: (i) studies written in the English language; (ii) studies that had undergone a standard peer-review process; and (iii) reports issued by governments or global health care organizations. Similarly, the exclusion criteria were as follows: (i) non-English studies; (ii) non-peer-reviewed studies; (iii) duplicate results; (iv) conference papers or proceedings; (v) studies that did not use standard measurement tools; (vi) studies without methodology or clearly explained findings; and (vii) reports from suspicious non-profit organizations (i.e., non-profit organizations without a clearly defined structure, funding sources, etc.) or studies published in “potentially” predatory journals (i.e., journals are not included in SCIE, that do not have clear review policy, have inappropriate ISSN, etc.). In addition to the inclusion and exclusion criteria, we considered only the most recent and relevant studies in each of the considered topics. Besides, when multiple studies of different authors evidencing similar findings were found, we included only those studies which were accepted first for the publication (i.e., the date of acceptance was considered). Our further in-depth comprehensive review of the selected studies (i.e., those satisfying the inclusion and exclusion criteria) included study objective(s), design, year of the publication, country where study was carried out, the sample size, the main findings, etc. Finally, the key findings of studies satisfying all the inclusion and exclusion criteria and other additional criteria are summarized in Tables 1–6.

## 3. Relationship between the Late Life Depression and Dementia (Alzheimer’s Disease)

Depression and dementia, which are the most common mental health problems in the elderly, have a complicated relationship that is not yet fully understood. We remind the reader that depression causes cognitive changes, while dementia is frequently accompanied by various mood symptoms [26,27]. Whether late life depression does or does not increase the risk of dementia, has become a fundamental question that is still undergoing extensive investigation. Most studies performed during past 30 years on depression and dementia are based on scores from existing self-report questionnaires used as depression screening tests [28,29,30,31]. The main limitation of these epidemiological studies is usually the relatively short follow-up period between the assessment of depression and the prodromes of dementia [31,32,33,34,35,36]. Some of these earlier investigations have suggested that the late life depression increases the risk of dementia and AD [37,38,39,40]. This conclusion was also supported by the well-known study of Ownby et al. [41], where, based on systematic meta-analysis and metaregression analysis, the authors found that individuals with a history of depression would be more likely to be diagnosed with dementia in later life. Evidences from other studies have, however, indicated that depression either has only a mild effect on dementia [42], does not increase the risk for development of dementia [43] or can be even the prodromal stage of dementia [44,45]. It is worth noting that severity and frequency of depression, differences in the sample sizes and types (e.g., women and men, education level), the culture and ethnical differences (e.g., Sweden, Finland, UK, USA, Australia, Japan, Taiwan), variation in used measures (e.g., operationalization and definition of depression) and different study lengths could be the main reasons for all these inconsistencies.

A majority of psychologists and psychiatrists agree that longer follow-up studies back at least into the midlife period are needed to clarify the relationship between a history of depression and the prodromes of dementia. In response, several long-term prospective cohort studies on depression and dementia have been recently performed [20,45,46,47,48]. Among them only a 17-years follow-up cohort study has indicated that depressive symptoms in older individuals can nearly double the risk for development of dementia and/or AD [45]. The main strength of this study is a well characterized cohort. Nevertheless, due to the unknown duration of the depressive symptoms, uneven patient adherence to depression treatment using antidepressants and no possibility of controlling other depressive factors such as the physical and social engagement of the individuals, which are discussed in details in the following section, the authors could still not eliminate the possibility that depression might be just the prodromal stage of dementia. Findings of other long-term cohort studies support the hypothesis that late life depression can be indeed the long and progressive prodromal symptom of dementia [20,47,48,49]. Especially, Singh-Manoux et al. [49] in their 28-year long follow-up study have proposed that depression and dementia share the same common risk factors. Since depressive symptoms such as a lack of motivation or depressed mood occur first, therefore they have suggested that these symptoms should be considered just the prodromal states of dementia and not its early triggers.

Although the additional long-term systematic investigations are still needed to support their conclusions, preliminary findings of these long-term cohort studies may already open a pathway for an early stage diagnosis of dementia, that is, an early stage diagnosis of dementia may require tracing the depressive symptoms back to the at least midlife. For readers’ convenience, the main findings of studies associating the late life depression with development of dementia and AD that are discussed in this section, are summarized in Table 1.

## 4. Other Established (Non-)Modifiable Risk Factors and Possibly Protective Factors and Their Association with Dementia and Alzheimer’s Disease

### 4.1. Association of Genetic Predisposition and Sex Hormones with Dementia and Alzheimer Disease

The risk for development of dementia increases exponentially with advancing age. As a result, majority of the oldest-old people is afflicted with at least one type of dementia such as Alzheimer’s disease or vascular dementia [50]. Age is, therefore, considered to be the strongest non-modifiable risk factor for development of dementia. Numerous clinical studies have proposed that a low level of apolipoprotein E (APOE) genotype [51,52,53] along with its genetic variations [54,55] increases the risk of dementia; especially for individuals where dementia has been diagnosed in first-degree relatives (see Table 2). The APOE, which is co-responsible for carrying various fats such as cholesterol through the bloodstream, plays an important role in regulation of the neurotoxic amyloid beta (Aβ) peptide. It alters the brain activity and metabolism (e.g., removal of amyloid plagues) and, correspondingly, a low level of APOE is then associated with the progress in cognitive decline (i.e., it enhances the structural and functional changes in brain that are known to be linked with an early sign of AD neuropathology). The higher risk for development of dementia may be also explained by interaction between the genetic and environmental factors [56]. It is worth noting that the association between a low APOE level and the higher risk of dementia has been recently supported by a Mendelian randomization study performed on two large population cohorts (106,562 and 75,260 individuals) [57].

Deposition of the neurotoxic amyloid beta and alteration of *t*-tau proteins levels that may impair the cognitive functioning of the individuals and subsequently lead to dementia are known to be long-term processes (i.e., alteration in cerebrospinal fluid has been recently observed even for individuals in the asymptomatic stage of AD [58]). Hence, it can be of great importance to perform long-term investigations on how the APOE level and cerebrospinal fluid changes in the early-, mid- and late-life are connected with a higher/lower risk for development of dementia. We emphasize here that this kind of studies would probably require, in addition to the standard psychiatric and psychological methods, the development of novel brain activity mapping techniques [67], along with mass spectrometry in gaseous or aqueous solutions [68].

Some studies have observed that the risk factors and progression of dementia are also gender- dependent. Females are generally more vulnerable to genetic and environmental risk factors than males, resulting in a higher prevalence of dementia in elderly women [59,69]. Moreover, it has been found that females are in a greater risk for development of AD, whereas males have a higher chance of vascular dementia. Sex difference as the risk factor for the development of dementia and the underlying mechanisms are not yet fully understood. It could probably be caused by a combination of several factors, including the developmental and physiological differences between males and females (e.g., the environmental stimuli and exposure to various hormones may create gender - sex differences in the brain structures) and lifetime sex hormone level changes (e.g., a significant drop of the estrogen level after menopause) [70]. Estrogen increases the cerebral blood flow and has the neuroprotective properties. It plays the important role in regulation of Aβ peptide against oxidative stress and neuroinflammation. As a result, estrogen has a beneficial effect on the cognitive functions and helps to improve the cognitive dysfunction observed in AD individuals. Surprisingly, effectiveness of the postmenopausal hormone-based therapy on delay the prodromes of dementia remains uncertain. While earlier population-based studies suggested that postmenopausal hormone therapy reduces the risk for development of dementia [60], the results of more recent studies have indicated that postmenopausal hormone therapy either does not reduce [61] or may even increase the risk of dementia [62]. The inconsistency in these findings may be explained by a significantly different effect of the hormone therapy timing after menopause. For example, about 30% lower chance for AD was observed for females using hormone therapy within 5 years of menopause, whereas for those using the hormone therapy after 5 or more years of menopause the opposite effect was found, that is, a higher risk for development of AD [63].

Testosterone, the male sex hormone, is neuroprotective and it contributes to the removal of Aβ peptide. Low testosterone levels are often associated with vascular problems, heart attacks, strokes or depression. Hence, elderly males with a low level of sex hormone are more vulnerable to cognitive impairment and, correspondingly, may be at a higher risk of dementia [64]. This hypothesis has been recently supported by a 10+ years follow-up cohort study performed on 4069 males [65]. Interestingly, some other earlier studies provided evidence associating low testosterone and an increased risk of dementia just in males with either a high education level or age over 80 [66]. It is expected that testosterone therapy might help to reduce the risk of dementia in elderly males. Unfortunately, an earlier clinical study has provided evidence supporting only a mild effect of testosterone treatment on cognitive function [71]. We can therefore easily conclude that a possible future studies topic should be to target the efficiency of hormone therapy (estrogen and testosterone) for different hormone therapy timing and duration, individuals’ education level, age, ethnicity, etc. The main findings of studies associating genetic predisposition and changes in the sex hormones with dementia considered in this review are summarized in Table 2.

### 4.2. Association of Depression, Obesity, Diabetes Mellitus and Blood Pressure with Dementia and Alzheimer Disease

Genetic predisposition cannot be notably modified by intervention or the behavioral changes of individuals. The interactions between genes, psychosocial and lifestyle or environmental factors are associated with a higher (lower) risk for later-life dementia. In past decades multiple protective and possibly modifiable risk factors were identified and their associations with dementia studied. A history of depression, obesity, hypertension, diabetes mellitus, dyslipidemia, cardiovascular diseases, sleep disturbances, brain injury, smoking and alcohol misuse are among the best-known modifiable risk factors. For example, some early studies have provided convincing evidence supporting an association between early life and midlife depression with an increased risk of dementia in later life [72]. The weakness of these studies is usually a relatively short follow-up period. In contrast, the results of recently published long-term longitudinal studies do not support an association between midlife depression and an increased risk of dementia [20,48]. Instead, they have considered depression to be the prodromal stage of dementia because they both share similar common neuropathological processes [49]. Recalling that a detailed discussion on the relationship between depression and dementia was presented in the previous section, the association between clinical anxiety in midlife and an increased risk for development of dementia has been also highlighted [73]. Whether clinical anxiety increases the risk of dementia or is just its prodromal stage is, nevertheless, currently still under extensive active investigation (i.e., in ongoing long-term follow-up studies with well-defined cohorts).

The number of individuals with obesity has nearly tripled in past 30 years and in most Western countries obesity has already become prevalent. It has been suggested that the inflammation and oxidative stress caused by obesity affect the brain in the same manner as known for the arteries and/or the inner organs, leading to the neurodegeneration and, subsequently, the development of dementia [74]. As a result, numerous studies have considered midlife obesity as a possible modifiable risk factor for the development of dementia [75,76,77]. We emphasize here that recent evidence from the results of a 15+ years long prospective study performed on an enormously large cohort of ~ 1.1 million individuals supports a strong link between midlife obesity and vascular dementia, whereas no such connection between obesity and AD has been observed [78]. Obesity itself is often associated with other possibly modifiable risk factors including diabetes mellitus or hypertension as shown in Table 3 [79]. Diabetes mellitus is a metabolic disorder characterized by insulin resistance or insulin deficiency. During diabetes variations in plasma osmotic pressure and oxidative stress levels may result in cognitive changes in the brain, that is, the imbalance between oxidants and antioxidants initiates neurodegeneration through the excessive deposition of Aβ peptide and the release of free radicals, and, correspondingly, they can lead to dementia [80,81]. The preliminary results of recent clinical trial studies have indicated that intranasal insulin therapy may help improve verbal memory, especially, the story recall performance of the APOE genotype [82]. We emphasize here that the main drawback of these studies, performed in one country (USA) is the small cohort, comprising of only 293 individuals. Thus, in order for the promising efficiency of intranasal insulin therapy in the treatment of dementia and AD to be confirmed, additional worldwide clinical studies on a large population cohort still need to be performed.

The cerebral circulation is strongly blood pressure dependent; therefore, the integrity of white matter can be easily impaired by structural changes or damage to the small arteries (e.g., microinfarcts) caused by high (low) blood pressure [90]. This immediately implies that chronic hypertension not only increases the risk for cerebrovascular disorders like cerebral infarcts but also impairs the brain cognitive functions, which in the worse situation could lead to vascular dementia [83]. More recently, an association between high blood pressure and AD has been suggested by numerous studies [83,84,90,91,92,93]. High blood pressure has been observed to cause an aggregation of the neurotoxic Aβ peptide in the brain that in time can lead to cerebrovascular disfunction and AD [94]. It has been also found that in contrast to vascular dementia, where an increased level of Aβ peptide is observed, the dementia level of AD is linked with a low level of Aβ peptide [85]. This strong impact of midlife chronic hypertension on the increased risk for dementia in later life has been supported by a 24-year follow-up study carried out on 4751 individuals [86]. Surprisingly, a protective function of high blood pressure in the oldest-old has been found for individuals with hypertension developed in late life [87]. The current hypothesis suggests that high blood pressure in the oldest-old compensates for their age-related vascular changes and, consequently, may help to maintain normal cognition. Gilsanz et al. [88] have studied the relationship between midlife hypertension and the prodromes of dementia in terms of sex difference. According their findings, midlife hypertension increases the risk of dementia for females only, while no association between midlife hypertension and dementia in male individuals has been observed. Regrettably, a sufficient amount of evidence supporting both the protective function of hypertension in the oldest-old and sex differences with the risk for development of dementia is still missing. In general, blood pressure can be easily controlled by various medications. Numerous studies have revealed no differences between individuals using anti-hypertensive medications and those without hypertension [89,93]. It has also been observed that the control of blood pressure during the lifetime, especially during sleep, can significantly decrease the risk for development of dementia in later life [84].

Table 3 clearly shows that the risk of cognitive impairment and dementia that is associated with obesity, diabetes (type 2) and hypertension, can be significantly reduced by a combination of a healthy diet and physical activities. Individuals with healthy dietary patterns, that is, a regular intake of fruits, vegetables, whole grains, nuts, and fish are in a reduced risk of later life dementia. The is due to a fact that a healthy dietary pattern with regular physical activity helps individuals keep their body mass index below the obesity level and improve their memory and attention, as well as the other cognitive functions. Future studies may consider investigating the effectiveness of different healthy diet and/or physical activity timing on a reduced risk for the development of dementia and AD in later life.

### 4.3. The Relationship between Dementia (Alzheimer’s Disease) and Education Levels (Social Engagement)

The brain reserve that enhances the individual’s resilience to prevent or delay the prodromes of dementia, is known to be associated with one’s level of education [95]. Plenty of studies have suggested that early life education stimulates the brain to build the cognitive reserve, therefore, higher education may help decrease the risk of later life dementia (see Table 4) [96,97,98]. For example, clear evidence supporting association of the higher education with a lower risk of dementia has been provided by Matthews et al. [99]. In their long-term (two-decades) comparative study earlier born elderly individuals are showing more dementia cases than those born later in the past century. The lower risk for development of dementia in the later born elderly can be explained by a combination of the multiple factors including improvements in the early stage diagnosis of cerebrovascular and heart diseases and the widespread support for early life education during the past century. The autopsy-verified findings from the Nun Study provide additional evidence supporting the beneficial effect of more education on the cognitive functions in later life, even for individuals with AD pathology [100]. The results of other studies have suggested that the non-modifiable genetic predisposition caused by a negative effect of APOE can be noteworthily suppressed in individuals with high education levels [101,102]. The establishment of firm connections between higher education levels and a delay in the prodromes of dementia still requires however further long-term studies.

Table 4 shows that the social engagement of elderly individuals can also play an important role in the prevention of dementia. Individuals with strong social engagement or positive social support from their family members and friends often have a lower risk of getting dementia in later life than those with negative social support or low social engagement [103,104,105,106,107]. The mechanism(s) underlying the connection between social engagement (social support) and the risk of dementia remain unclear, although several hypotheses to explain their relationship have already been proposed. First, social engagement may stimulate cognitive function and help maintain the brain reserves needed to prevent or delay the prodromes of dementia [95,110]. Second, this engagement improves the immune system and supports the central nervous system to preserve its internal stability [108]. Positive social support can, particularly, help significantly reduce chronic stress, which is a well-known risk factor for the development of AD, that is, chronic stress causes functional changes in the microglia immune cells, which are co-responsible for the removal of toxic materials from the brain [111]. Third, people with good social engagement are much less vulnerable to other risk factors such as smoking, alcohol misuse, obesity or depressive mood [104]. Some other studies have addressed the role of sex differences in the association between social engagement and the risk of dementia. For instance, according the findings of a recent 10-year follow-up study, social engagement decreases the risk of dementia just for male individuals, while for females no any significant effect of social engagement on dementia has been found [109]. Despite its large population sample (*n* = 14,088), this study does not account for the effect of cultural differences (the study was performed just in Japan, where social interactions within the community are significantly different from those known in Western countries), therefore it has been suggested that studies performed in different culture societies would be needed to confirm the general validity of the findings.

We note that in past 30 years we have been witnesses to great advances in communication technology, which have enabled the establishment of new ways of social engagement through the so-called instant messengers, social networking applications or e-mail. As a result, some researchers hypothesize that this novel communication technology may be considered as an alternative way of maintaining cognitive health, particularly for the elderly who are living alone and/or without the family support [112]. Whether the novel communication technology can or cannot delay cognitive decline and, subsequently, help reduce the risk of development of dementia, may be considered as one of the research goals of future studies associating social engagement with dementia.

### 4.4. The Relationship between Dementia (Alzheimer’s Disease) and Various Drugs

The acute use of alcohol, legal or illegal drugs like benzodiazepines, cannabis or tobacco smoking may impair individuals’ cognitive functions and increase the risk for the development of dementia in later life. The findings of numerous studies have suggested that not only heavy drinkers but also abstainers (the two extreme limits) are at higher risk of later life dementia, whereas individuals consuming light-to-moderate amounts of alcohol may be at lower risk of dementia [113]. The protective effect of light-to-moderate alcohol intake has been supported by a recent 23-year long follow-up study, in which the cohort has been followed back from the middle age [114]. The same effect has been also observed in elderly populations, where moderate drinking may be associated with a lower risk of dementia death than among abstainers [115]. It is not surprising that published studies evaluate the alcohol consumption level based on self-report measures. These studies do not account for the different culture “standards”, that is, the meaning of heavy, moderate and light levels of alcohol consumption differs worldwide, and, correspondingly, the obtained findings and conclusions might be easily mispresented. Moreover, alcohol consumption is regarded as a priority issue related to public health and studies investigating associations between alcohol consumption and dementia are either not easy to perform or even restricted. Hence, without a clear understanding of the biological mechanisms underlying the connection between alcohol consumption and the risk of dementia, no conclusions on alcohol drinking can be drawn yet. It is important to note that another recent study provides evidence associating a higher risk of dementia even with the light-to-moderate alcohol consumption [116]. The key findings of the presented studies on alcohol consumption and dementia are summarized in Table 5.

In contrast to alcohol misuse, where the findings on the effect of alcohol consumption levels on the risk of dementia are inconsistent, smokers always have a higher risk of dementia in later life (see Table 5). Cigarette smoking produces oxidative stress in the brain [117,120] that in time can cause vascular disfunction and lead to dementia. Smokers are generally more vulnerable to cardiovascular events and vascular dementia than non-smoking individuals. Surprisingly, no strong evidence supporting a connection between passive smoking and an increased risk of dementia has been yet found [118]. On the other hand, the risk of dementia is reduced once an individual quits smoking for a prolonged time, therefore, it is of great importance to public health to help smokers quit smoking [119].

### 4.5. Relationship between Sleep Disorders and Dementia (Alzheimer’s Disease)

Individuals with vascular dementia, AD and other types of dementia are often diagnosed with sleeping pattern problems. Many studies, which findings are given in Table 6, have proposed an association between sleep problems, especially, for obstructive sleep apnea patients and a higher risk of dementia [56,121,122,123]. Some researchers believe that obstructive sleep apnea may even start the AD neuropathological process [124]. In general, sleep problems cause changes in Aβ peptide and *t*-tau protein levels, and probably may even complicate the removal of neurotoxic Aβ peptide [118]. Importantly, a connection between sleep behavior disorders and dementia is currently supported by the findings of a long-term follow-up study [125]. Unfortunately, results supporting the protective effect of sleep medication on a reduced risk of dementia are yet inconsistent [126]. For example, the frequent use of sleep medication in midlife may increase the risk of dementia. In contrast, treatment of obstructive sleep apnea by continuous positive airway pressure enables one to reduce sleep fragmentation and, afterward, helps stabilize levels of Aβ peptide *t*-tau protein as well as improve the cognitive functions of individuals diagnosed with obstructive sleep apnea [127]. Some studies have also suggested to use of cannabinoids in the treatment of dementia [128]. However, studies focusing on the effectiveness of cannabinoids and/or sleep medication for the treatment of dementia are currently under extensive investigation and the results of these clinical studies are still not available.

## 5. Future Possible Research Directions and Trends in the Treatment of Dementia and Alzheimer’s Disease Using Single-Domain and Multi-Domain Interventions

The vulnerability of individuals to cognitive decline and an increased risk of dementia or AD is complex. It depends, in addition to age and genetic predisposition (non-modifiable risk factors), on several protective and potentially modifiable risk factors. Hence, it is generally accepted by psychologists and psychiatrists that the cognitive functions can be maintained and the risk for cognitive decline and, correspondingly, the development of later life dementia and AD can be notably reduced by interventions targeting individuals’ risk factors [21]. These interventions may target one given dementia risk factor, often referred as the “single-domain” interventions, or multiple risk factors of dementia, also known as “multidomain” interventions. Despite the fact that a large number of potential single-domain interventions were already identified and we also briefly discussed some of the possible interventions in previous section, there is still a lack of supporting evidence from randomized control trials for the effectiveness of these interventions. We also emphasize here that the results of recent long-term follow-up studies support the hypothesis that the prodromes of dementia may possibly start more than decade before any clinical symptoms can be observed [46,47,48]. The short duration of treatment (trials) and small sample size are among the main limitations of the most published randomized control trials [129]. Other limitations can even include a not well-defined cognitive outcome. We only note that many researchers are using different measures, sample sizes and terminologies, complicating the interpretation and generalization of their findings. As a result, existing trial studies usually do not provide sufficient information on what intensity of intervention targeting a given risk factor is needed to preserve cognitive function. We can, therefore, conclude that a possible future research direction could be targeting the relationship between the intensity of a given intervention and cognitive functioning. Nevertheless, before any systematic trials can be carried out, a precise terminology should be established [8]. Then, the large sample size and a long follow-up period that might go back even far beyond the midlife with clearly defined trial conditions would be required to find the relationship(s) between various interventions and a possibly reduced risk of dementia and AD.

The neuropathological processes that may cause the cognitive decline and lead to dementia and AD depend on the genetic predisposition, age and the combination of multiple possibly modifiable risk factors (discussions on known possibly modifiable risk factors are given in Section 3 and Section 4) [130]. We remind the reader that, for instance, midlife obesity is often associated with hypertension and diabetes mellitus. These three possibly modifiable risk factors can cause an accumulation of neurotoxic peptides and oxidative stress level changes in the brain that in time may result in cognitive impairment and dementia [78,81,89]. Similarly, the higher risk of dementia in the elderly individuals with various comorbid cardiometabolic diseases such as diabetes mellitus or stroke can be moderated by social engagement and physical activities [131]. The effects of (non-)modifiable risk and protective factors are also age- and gender-dependent. As an example, we can consider hypertension, which during the midlife increases the risk of later life dementia but hypertension developed in late-life may have the protective effect [92]. Since multiple risk and protective factors usually occur together and their effect on the individual’s cognitive functions may be gender dependent and varies in time, therefore single-domain interventions might not be sufficient to maintain cognitive functioning and delay the prodromes of dementia for a majority of the so-called “at-risk” individuals. Multidomain interventions targeting multiple risk factors may be of importance to preserve or improve individuals’ cognition and reduce the risk for development or delay the prodromes of dementia [132]. One of the earliest trials performed on a large sample size comprised of 1260 individuals provided evidence supporting the effectiveness of multidomain interventions in the prevention of dementia for at-risk individuals [133]. In contrast, some other earlier multidomain trials did not meet the expected outcome criteria (a decrease in the overall number of dementia cases) but they provide evidence, even though still limited, supporting improvements for individuals with untreated hypertension or complications with the removal of amyloid peptide from the brain [134,135]. Future multidomain interventions should, therefore account for the differences and heterogeneity in the risk factors of individuals, culture, and ethnical differences, etc. Then, each group of people with the similar risk factors at a given time-period, culture background and the social engagement should be treated by multidomain interventions specially designed for a given group of individuals [136]. The electronic health (eHealth) and mobile health (mHealth) solutions can also help design more efficient multidomain trials and provide support for “at-risk” elderly individuals [137]. Personalized target multidomain interventions may be, therefore, considered as one of the future trends in prevention and treatment of dementia.

Based on the evidence discussed in Section 3 and Section 4, we may also hypothesize that to understand the underlying mechanisms of dementia (and AD) neuropathology would probably require tracing individuals’ multiple risk and protective factors back to at least their midlife or even to early life (e.g., individuals with higher idea density scores in early life were found to not have cognitive deficits in later life [138]). In addition, these future studies may also need to link changes in body fluids during an individual’s lifetime and subject to various risk factors (i.e., each risk and protective factor may affect individual differently in early-, mid- or later life). We also envisage that changes in body fluids in early to midlife may be associated with different roles of individual risk factors and, correspondingly, with the risk for development of dementia and AD in later life. This hypothesis, however, requires future systematic investigation, that is, multiple observational studies and clinical trials to be carried out.

### Limitations

The main limitation of this literature review is the lack of a systematic analysis of the published literature review (i.e., we include the latest studies that are rigorous, with results clearly supported by data, and measures that are well established. We excluded non-English studies, duplicate results, studies where data are not clearly explained, or the hypotheses are not supported by evidence/data). Details of the inclusion and exclusion criteria are given in Section 2. However, dementia and AD are heterogeneous disorders caused by a combination of the multiple (non-)modifiable factors (e.g., late life depression, hypertension, obesity, social engagement, etc.). Hence, to understand the underlying mechanisms of dementia and AD pathogeneses requires their multifactorial nature represented by the combination of the multiple factors to be considered. We therefore believe that it is of great value to the clinician and public health audience to provide a literature review focusing on the latest understanding of the roles of the individual identified (non-)modifiable risk factors in the development of dementia and AD, rather than focusing on any particular risk factor.

## 6. Conclusions

This review presents a literature overview of the latest findings associating known (non-)modifiable risk factors with the risk for development of dementia and AD. The advantages, drawbacks and limitations of possible interventions have been also discussed. Furthermore, possible future directions in research and treatment of dementia are highlighted. Overall, this review accounts for the latest suggestions, hypothesis, and findings in the fast-growing field of dementia and AD that can be used to design the future strategies needed for prevention and treatment of both dementia and AD.

## Figures and Tables

**Table 1:** Main findings of studies associating the late life depression and dementia (Alzheimer’s disease).

Study	Sample Size/Study Length	Measures/Design Questionnaire	Diagnosis/[OR/HR] Depression Status
Van Wanrooij, et al. [26]	3299 (F: 1490; M: 1490)/6.7 Years	Geriatric Depression Scale apathy (GDS-3A); 12 depression (GDS-12D); The AMC Linear Disability Score	VAD/not discussed
Balsis et al. [27]	108/5.26 Years (3.68; 7.97; 4.12)	Blessed Dementia Scale; Five-factor model of personality	AD/not discussed
Barnes et al. [29]	2220 (F: 1319; M: 901)/6 Years	MCI; MMSE; MRI; Digit Symbol Test, Benton Visual Retention Test, Telephone Interview; Center for Epidemiological	VAD & Cardiovascular disease/Moderate Depressive OR: 1.37 [95% CI = 1.00–1.88]; High Depressive OR: 2.09 [95% CI = 1.46–2.97] Associated with MCI
Johnson et al. [30]	2963/length not specified	Neuropsychiatric Inventory Questionnaire	AD: 2474; Parkinson Disease dementia: 74; Dementia with Lewy bodies: 151; VAD: 85-
Leoutsakos et al. [31]	328 (F: 216; M: 112)/3.7 Years	MMSE; Clinical Dementia Rating	AD/not discussed
Gatz et al. [32]	766 (F: 511; M: 255)/5 Years	CES-D, participant-reported medical history, and duration of depression	AD/Adjusted OR: 1.50 [95% CI = 0.49–4.63]; Dementia OR:1.84 [95% CI = 0.76–4.47]
Geerlings et al. [34]	3147 (F: 1929; M: 1218)/3.2 Years	CES-D; MMSE	AD/Adjusted OR:1.67 [95% CI = 0.76–3.63]
Burke et al. [36]	12,053 (F: 7865; M:4188)/10 Years	Clinical Rating Scale	AD/HR: 2.32 [95% CI, 1.87–2.88]
Barca et al. [38]	282 (F: 152; M: 130)/2 Years	Cornell Scale for Depression in Dementia; Clinical Dementia Rating Scale; MRI; MMSE.	AD/Depressive symptoms on the Cornell Scale average probabilities above 0.80
Kaup et al. [40]	2488 (F:1332; M: 1156)/1 Year	Center for Epidemiologic Studies Depression Scale Short Form (CES-D-10); Modified MMSE	Dementia/HR: 1.94 [95% CI = 1.30–2.90]
Ownby et al. [41]	102,172 (20 studies)/length not specified	Meta-analysis & meta-regression analysis	AD/Coefficient, 0.003; SE, 0.001; z = 2.01; *p* = 0.05
Tapainen et al. [42]	27,948 (F: 18,934; M: 9014)/43 Years (medical records from 1972)	ICD-8, ICD-9 and ICD-10	AD/5-year AD adjusted OR = 1.17, [95% CI = 1.05–1.30]; 10-years adjusted OR = 1.08, [95% CI = 0.96–1.23]
Becker et al. [43]	288/7.1 Years	CES-D; Modified MMSE	AD/Persistent depression HR: 1.33 [95% CI = 0.49–3.65]; Transient depression HR: 1.62 [95% CI = 0.78–3.35]
Brommelhoff et al. [44]	547 (F: 372; M: 175)/4 Years	TELE cognitive screening instrument; Blessed Dementia Rating Scale	Dementia/OR:1.72 [95% CI = 1.07–2.76]
Saczynski et al. [46]	164 (F: 104; M: 60)/17 Years	CES-D	Dementia & AD/Dementia HR: 1.72 [95% CI = 1.04–2.84]; AD HR: 1.76 [95% CI = 1.03–3.01]
Li et al. [47]	685 (F: 408; M: 250)/15 Years	Cognitive Abilities Screening Instrument; CES-D	Dementia/Late life depression: HR: 1.46 [95% CI = 1.16–1.84]; Early life depression: HR: 1.10 [95% CI = 0.83–1.47] history of depression early-life (<50 years)
Amieva et al. [48]	350 (F: 242; M: 108)/14 Years	Isaacs Set Test (IST); Benton Visual Retention Test; MMSE; Wechsler Similarities test; Instrumental activities of daily living	AD/not discussed
Singh-Manoux et al. [49]	10,189 (F: 3351; M: 6838)	30-item General Health Questionnaire (GHQ-30); CES-D	Dementia/Those reporting depressive symptoms in 1985 HR: 1.21 [95% CI = 0.95–1.54]; Those reporting depressive symptoms in 2003 HR: 1.72 [95% CI, 1.21–2.44]

OR—Odds ratio; HR—Hazard ratio; CI—Confidence interval; F—Female; M—Male; MMSE—Mini- Mental State Examination; CES-D—Center for Epidemiologic Studies Depression; AD—Alzheimer’s disease; VAD—Vascular dementia; MCI—Mild cognitive impairment.

**Table 2:** Effect of the genetic predisposition and changes in sex hormones levels on the risk of dementia/AD.

Study	Factor	Effect/Findings	Interventions
Kivipelto et al. [52]	APOE ε4	Risk of dementia (AD) OR: 2.83, 95% CI = 1.61–4.97	Lifestyle improvements such as physical activity, low dietary fat intake, reduced alcohol consumption and quit smoking => reduces risk of dementia and AD
Licher et al. [55]	APOE gene	Dementia risk in individuals with the unfavorable profile is higher than of those with the favorable one (HR: 2.51, 95% CI = 1.40–4.48 vs. HR: 1.39, CI = 1.04–1.85); for those at high APOE risk – no differences in dementia risk between those with unfavorable/intermediate profiles compared to those with favorable profile (HR: 1.00, 95% CI = 0.79–1.28 and HR: 1.05, 95% CI = 0.731.50)	Same as suggested in Kivipelto et al. [50]
Burke et al. [56]	APOE gene-environment interactions	AD risk is significantly higher for those with APOE genotype combined with sleep disturbance/depression. APOE ∈4 & Recent depression (HR: 8.15) & clinician-verified depression (HR: 10.11); sleep disturbance (HR: 6.79).	Possible alteration of depression and sleep disturbance may help reduce risk of AD
Rasmussen et al. [57]	APOE gene-environment interactions	Lower APOE level is associated with increased risk of Alzheimer disease and all dementia, in accordance with the observational associations. Risk of Alzheimer disease and all dementia increased stepwise with stepwise lower APOE levels.	Not discussed
Johnson et al. [58]	APOE gene-environment interactions	The sugar metabolism module showed the strongest AD trait correlations.	Not discussed
Farrer et al. [59]	APOE gene with account for age, sex, etc.	AD was significantly increased for Caucasian with genotypes ∈2/∈4 (OR = 2.6, 95% CI = 1.6–4.0), ∈3/∈4 (OR = 3.2, 95% CI = 2.8–3.8), and ∈4/∈4 (OR = 14.9, 95% CI = 10.8–20.6); Weaker (stronger) effect of ∈4 observed for Hispanics and Afro-Americans (Japanese); ∈2/∈3 genotype protective across all ethnic groups	Not discussed
Waring et al. [60]	Sex hormones (Estrogen)	The inverse association between estrogen therapy and AD remains significant even after adjustment for education and age at menopause	Postmenopausal ERT => reduced risk of dementia
Imtiaz et al. [61]	Sex hormones (Estrogen)	No effect on risk of AD in register-based data [HR: 0.92 (0.99), 95% CI = 0.68–1.2(0.75–1.3)] & Long-term self-reported postmenopausal hormone therapy association with a reduced AD risk (HR: 0.53, 95% CI = 0.31–0.91)	Postmenopausal hormone therapy has no effect on AD & Long-term self-reported hormone therapy can reduce risk of AD
Savolainen-Peltonen et al. [62]	Sex hormones (Estrogen)	Risk of AD was increased but did not differ significantly between users of estradiol (OR = 1.09, 95% CI = 1.05–1.14) and those of oestrogen- progestogen (OR = 1.17, CI = 1.13–1.21). Exclusive use of vaginal estradiol was not related to risk of AD (OR = 0.99, CI = 0.96–1.01).	Use of vaginal estradiol - Long term use of systemic hormone therapy may increase risk of AD
Shao et al. [63]	Sex hormones (Estrogen)	Hormone therapy and reduced risk of AD depend on timing of use. Hormone therapy started within 5 years of menopause associated with significantly lower AD risk (HR: 0.70; 95% CI = 0.49 – 0.99) & Hormone therapy started later than 5 years showed no such association (HR:1.03; 95% CI = 0.68–1.55).	Early (in 5 years) use of hormone therapy => reduces risk of AD; Late (more than 5 years) use of hormone therapy => increased risk of AD
Lv et al. [64]	Sex hormones (Testosterone)	Low plasma testosterone level is associated with an increased risk of AD in elderly men (random RR = 1.48, 95 % CI = 1.12–1.96); Low testosterone increase risk for impairing cognitive function in the elderly men	Testosterone treatment for males with a low testosterone level may prevent or delay the prodromes of AD.
Ford et al. [65]	Sex hormones (Testosterone)	Risk of dementia increases with decrease of testosterone level (HR: 1.14, 95% CI = 1.03–1.26)	Testosterone treatment may improve cognition or prevent further cognitive decline.
Carcaillon et al. [66]	Sex hormones (Testosterone)	No significant association was found between Total-17-b estradiol and dementia/low testosterone level increases risk of dementia in men	Not discussed

OR—Odds ratio; HR– Hazard ratio; CI – Confidence interval; RR – Relative risk; AD – Alzheimer’s disease.

**Table 3:** Association of the obesity, diabetes mellitus and hypertension with the risk for development of dementia/Alzheimer’s disease.

Study	Factor	Effect/Findings	Interventions
Xu et al. [75]	Obesity	Overweight-OR = 1.71, 95% CI = 1.30–2.25 and obesity-OR = 3.88, 95% CI = 2.12–7.11	Healthy diet and physical activities to reduce BMI in midlife to normal level reduces risk of later life dementia/AD.
Albanese et al. [76]	Obesity	Midlife (age 35 to 65 years) obesity (BMI≥30) (RR = 1.33; 95% CI = 1.08–1.63) associated with dementia in late life	Same as suggested in Xu et al. [75]
Kivimaki et al. [77]	Obesity	per 5-kg/m^2^ increase in BMI for dementia was assessed before dementia diagnosis 10 years (HR:0.71, 95% CI = 0.66–0.77); 10–20 years (HR:0.94, 95% CI = 0.89–0.99); >20 years (HR:1.16, 95% CI = 1.05–1.27)	Not discussed
Floud et al. [78]	Obesity	Dementia detection during years 15+ was associated with baseline obesity (BMI 30+ vs. 20–24 kg/m^2^: RR = 1.21, 95% CI = 1.16–1.26, *p* < 0.0001)	Same as suggested in Xu et al. [75]
Fan et al. [81]	Diabetes mellitus	Diabetes mellitus individuals are having adjusted HR: 1.47, 95% CI = 1.30–1.67, *p* < 0.001), Diabetes mellitus increases notably risk of dementia	Prevention of comorbidities (e.g., hypertension and hyperlipidemia) during diabetes reduces risk of dementia
Avgerinos et al. [82]	Diabetes mellitus	Intranasal insulin improves verbal memory, its effect differs for apoe4 allele carrier status (i.e., apoe4(-) stronger cognitive gain than apoe4(+))	Intranasal insulin intervention reduces risk of dementia
Emdin et al. [83]	Hypertension	Link between usual systolic blood pressure and risk of vascular dementia decreases with age (per 20 mm Hg higher systolic blood pressure, HR:1.62; 95% CI = 1.13–2.35 at 30–50 years; HR:1.26, CI = 1.18–1.35 at 51–70 years; HR:0.97, CI = 0.92–1.03 at 71–90 years)	Control blood pressure to reduces the risk of dementia
Nagai et al. [84]	Hypertension	Antihypertensive therapy may reduce the risk of dementia by i) 11% (OR = 0.89; 95%CI = 0.69–1.16) or ii) (HR: 0.87; 95% CI = 0.76–1.00)	Blood pressure control during sleep has a neuroprotective effect on the brain, it prevents the incidence of dementia.
Janelidze et al. [85]	Hypertension/Plasma *β*-amyloid	Higher plasma level Aβ is linked with hypertension; For AD patients the levels of Aβ40 and Aβ42 were reduced & Preclinical or prodromal stage of AD linked with low Aβ42/Aβ40 ratio and/or Aβ42 plasma level.	Not discussed
Walker et al. [86]	Hypertension	Sustained midlife to late-life hypotension & midlife hypertension and late-life hypotension increases risk for subsequent dementia (HR:1.62, 95% CI = 1.11–2.37])	Not discussed
Corrada et al. [87]	Hypertension	Hypertension onset age 80 to 89 – lower dementia risk (HR: 0.58; Onset age of 90+ lowest dementia risk (HR:0.37); Developing hypertension at older ages may protect against dementia	Not discussed
Gilsanz et al. [88]	Hypertension	Mid-life hypertension associated with ~65% increased dementia risk only among women but not men. [95% CI = 1.25–2.18]	Hypertension treatment reduces risk of dementia
Murray et al. [89]	Hypertension	Individuals prescribed any antihypertensive medication have a significantly reduced risk of dementia (HR:0.57, 95% CI = 0.37–0.88) compared to untreated hypertension	Antihypertensive medication-Control of blood pressure reduces risk of dementia

OR—Odds ratio; HR—Hazard ratio; CI—Confidence interval; RR—Relative risk; AD—Alzheimer’s disease.

**Table 4:** Relationship between the education level/social engagement and the risk for development of dementia or Alzheimer’s disease.

Study	Factor	Effect/Findings	Interventions
Skoog et al. [95]	Education & Hypertension	Education (OR = 0.70; 95% CI = 0.51–0.96); stroke (OR = 3.78; 95% CI = 2.28–6.29); Higher education increases cognitive reserve and reduces risk of dementia	Promote the higher education, which helps to build the cognitive reserve needed to reduce the risk of dementia
Xu et al. [96]	Education	Low education shows a more significant increment of dementia or AD risk (for dementia HR:1.81; 95 % CI = 1.59–2.06 & for AD HR:1.78; 95 % CI = 1.43–2.22); One year of the additional education reduces risk of dementia by ~7%.	Same as suggested in Skoog et al. [95]
Nguyen et al. [97]	Education	There is ~1.1% reduction in dementia risk per year of schooling (95% CI = 2.4–0.02).	Same as suggested in Skoog et al. [95]
Then et al. [98]	Education	Education years ≤10 years => Dementia free 70.4%; Education years >10 years 82.4%; => Protective effect of more years of education on a lower dementia risk with critical threshold of completing >10 years of education	Same as suggested in Skoog et al. [95]
Wang et al. [101]	Education and Apolipoprotein *ε*4	High education (8 years and more) was related to a lower dementia risk (OR = 0.5; 95% CI = 0.3–0.6); Higher education buffers the negative effect of APOE 4 on dementia occurrence	Same as suggested in Skoog et al. [95]
Appiah et al. [102]	Education and Apolipoprotein *ε*4	Interaction between education and APOE found; HR of mortality of ε4 carriers vs. non-carriers => i) HR:1.59, 95% CI= 0.64–3.96 (post)graduate level; HR:6.66, 95% CI = 1.90–23.4 college level, HR:14.1, 95%, CI = 3.03–65.6 basic or high school level; Higher education weakens the adverse effect of *ε*4 on mortality	Same as suggested in Skoog et al. [95]
Zhou et al. [103]	Social Engagement	Social engagement association with dementia: low social engagement (OR = 0.71, 95% CI = 0.63–0.81); high social engagement (OR = 0.14, 95% CI = 0.06–0.28) and increased social engagement (OR = 0.33, 95% CI = 0.23–0.48) => participants with high or increased social engagement have lower risk of dementia	Maintain social engagement in elderly to reduce risk of dementia
Saito et al. [104]	Social Engagement	Being married, having social support of family members or friends, joining community groups, or engaging in work reduce risk of dementia	Same as suggested in Zhou et al. [103]
Khondoker et al. [105]	Social Engagement	Positive social support from children significantly reduced risk of dementia (HR:0.83, 95% CI = 0.69–0.99), while negative support increases risk of dementia (HR:1.31, 95% CI = 1.05–1.64).	Same as suggested in Zhou et al. [103]
Kuiper et al. [106]	Social Engagement	Low social participation (RR=1.41, 95% CI = 1.13–1.75), less frequent social contact (RR = 1.57, 95% CI = 1.32–1.85), and loneliness (RR = 1.58, 95% CI = 1.19–2.09) increase risk of dementia. Low social engagement has comparable effect on risk of dementia as observed for low education, physical in-activities, and depression.	Same as suggested in Zhou et al. [103]
Penninkilampi et al. [107]	Social Engagement	Poor social network (RR = 1.59, 95% CI = 1.31–1.96) & poor social support (RR = 1.28, 95% CI = 1.01–1.62). In long-term studies (≥10 years), good social engagement - modest protection (RR = 0.88, 95% CI = 0.80–0.96) and poor social engagement associated with increased dementia	Combat social isolation and provide support to older individuals who are lack of social engagement
Salinas et al. [108]	Social Engagement	Reduced risk of dementia in participants with stronger social network (HR:0.67, 95% CI = 0.49–0.92) and greater emotional support (HR:0.69, 95% CI = 0.51–0.94)	Same as prosed in Penninkilampi et al. [107]
Murata et al. [109]	Social Engagement	Family support on incident dementia beneficial only for men (HR:0.95 95% CI = 0.91–0.99); Family support for women (HR:1.00 95% CI = 0.97–1.04)	Promotion of social interaction with family members reduces risk of dementia

OR—Odds ratio; HR—Hazard ratio; CI—Confidence interval; RR—Relative risk; AD—Alzheimer’s disease.

**Table 5:** Studies associating addictive substances and cigarette smoking with the risk of dementia/Alzheimer’s disease.

Study	Factor	Effect/Findings	Interventions
Xu et al. [113]	Alcohol consumption	For individuals bellow age of 60 - Modest alcohol consumption (≤12.5 g/day) linked with a reduced risk of dementia with 6 g/day of alcohol, heavy drinking (~23 drinks/week or ≥38 g/day) increases risk of dementia.	Not discussed
Sabia et al. [114]	Alcohol consumption	Abstinence and heavy drinking in midlife were both associated with a higher risk of dementia.	Not discussed
Ormstad et al. [115]	Alcohol consumption	For individuals from 60 to 80, the abstinence is linked with a higher risk of dementia related death, while light alcohol drinking reduces risk (HR: 1.33, 95% CI = 1.14–1.56).	Not discussed
Dikalov et al. [117]	Tobacco smoking	Tobacco smoking enhances hypertension, induces mitochondrial oxidative stress, and enhance endothelial disfunction	Mitochondria-targeted interventions may help to improve changes caused by smoking
Batty et al. [118]	Tobacco smoking	Smoking increases risk of dementia => Plasma cotinine (HR:1.29, 95% CI = 1.05–1.59) and salivary cotinine (HR:1.10, 95% CI = 0.89–1.36)	Not discussed
Choi et al. [119]	Tobacco smoking	Risk of dementia for no smokers (HR:0.86, 95% CI = 0.75–0.99) and quit smoking long-time ago (HR: 0.81; 95% CI = 0.71–0.91) => quit smoking reduces risk of dementia	Help and encourage smokers to quit tobacco smoking

OR—Odds ratio; HR—Hazard ratio; CI—Confidence interval; RR—Relative risk; AD—Alzheimer’s disease.

**Table 6:** Studies associating sleep disorder with dementia and Alzheimer’s disease.

Study	Factor	Effect/Findings	Interventions
Elias et al. [121]	Sleep disorder-(OSA)	OSA causes an increased amyloid β -protein deposition and increases risk of AD. BMI & APOE ɛ4 can moderate β-amyloid deposition.	Not discussed
Przybylska-Kuć et al. [122]	Sleep disorder-(OSA)	Aβ40 level is much higher in patients with severe OSA than those with moderate OSA or no OSA, Higher Aβ40 may lead to AD	Not discussed
Hahn et al. [123]	Sleep disorder - (OSA)	Reduced sleep associated with ~75% increased risk of dementia (HR:1.75; 95% CI = 1.04–2.93) and doubles risk of AD (HR:2.01; 95% CI = 1.12–3.61)	Not discussed
Liguori et al. [124]	Sleep disorder - (OSA)	Sleep disruption and intermittent hypoxia induce orexinergic system and cerebral β-amyloid metabolism dysregulation and alteration in CFS orexin level, it supports hypothesis that OSA may start AD neuropathological processes.	Not discussed
Marchand et al. [125]	Sleep disorder – REM sleep disorder	REM sleep behavior disorder causes cognitive decline and is associated with neurodegenerative disorders that may lead to dementia	Not discussed.

OSA—Obstructive sleep apnea; HR—Hazard ratio; CI—Confidence interval; AD—Alzheimer’s disease.
